# The Effect of Online-Delivered Guided Imagery Relaxation on Stress and Well-Being of Primary School Children

**DOI:** 10.21315/mjms2023.30.4.10

**Published:** 2023-08-24

**Authors:** Chean Wei Lim, Azizah Othman, Hairul Anuar Hashim

**Affiliations:** 1 Department of Neurosciences, School of Medical Sciences, Universiti Sains Malaysia, Kelantan, Malaysia; 2 Department of Pediatrics, School of Medical Sciences, Universiti Sains Malaysia, Kelantan, Malaysia; 3 School of Health Sciences, Universiti Sains Malaysia, Kelantan, Malaysia

**Keywords:** mindfulness-based online intervention, guided imagery relaxation technique, stress, well-being, primary school children, randomised controlled trial

## Abstract

**Background:**

In the context of the coronavirus (COVID-19) pandemic, effectively coping with daily stressors is crucial for children who experience restrictions on physical movement and social activities. We examined the effects of the online-delivered guided imagery relaxation (GIR) technique on the stress and well-being of primary school children.

**Methods:**

Thirty-four (*N* = 34) 11-year-old students were randomly assigned to either intervention (*n* = 17) or waitlist-control (*n* = 17) groups. The participants from the intervention group were required to attend a 4-week online GIR session and practice the techniques daily for 5 min–10 min. The Stress in Children (SiC) questionnaire, the Strengths and Difficulties Questionnaire-Parent Reported (SDQ-PR) and tracking form were administered pre- and post-intervention.

**Results:**

Eleven (64.7%) participants attended all sessions and eight (47.0%) completed daily practices. Mixed-model ANOVA indicated no significant difference between participants from intervention and waitlist-control groups across pre- and post-intervention time points, with *P*-values greater than 0.05 for stress and well-being.

**Conclusion:**

Issues regarding online intervention, including managing children’s activities from a distance, Internet connectivity, and time limitations might have affected their adherence and the research outcome. Nonetheless, the online-delivered GIR technique is a promising intervention modality. However, its implementation should be improvised to be more impactful.

## Introduction

Emotional and behavioural problems, commonly reported in children and adolescents in contemporary society, can be categorised as internalising and externalising problems. Internalising problems refer to the emotional maladaptive responses, such as depression and anxiety, to cope with stress. Externalising problems refer to dysfunctional behaviours—being aggressive, defiant, or harming self and others (1, 2). Emotional and behavioural problems affect 10%–20% of all children worldwide; the number is 12.1% in Malaysia (3, 4).

If these problems are left untreated, children’s quality of life and well-being may be compromised and negatively impacted (5, 6). This is especially critical in the case of primary school children, who are 11 years old–12 years old and are transitioning into adolescence, a period marked with simultaneous critical physical, psychological and social development (7). They experience rapid biological and cognitive changes that may cause them to challenge and question their own socio-emotional and identity development (8). Hence, it is essential to introduce effective coping strategies to help them deal with negative affective and behavioural experiences (9–11). Consequently, children can be more resilient and well-prepared to manage adverse events in their adolescence or later stages of life, especially in the context of the COVID-19 pandemic (6, 12).

Mindfulness-based approaches have been widely applied to improve mental health and promote well-being among adult populations for the last 30 years (13, 14). Despite abundant empirical support for mindfulness-based techniques across different adult populations, the question of whether mindfulness exercises provide equivalent benefits for children remains unclear (15–17). Nevertheless, a few recent studies have revealed promising results of mindfulness-based interventions in enhancing psychosocial, cognitive, and behavioural aspects in elementary school children (18–20). More specifically, mindfulness-based interventions are effective in improving children’s psychological well-being, including their emotional functions, executive functions, attentional control, resilience, self-regulation and prosocial behaviour (21–25).

Among the various mindfulness-based interventions, the guided imagery relaxation (GIR) technique has attracted significant academic attention in recent years (26–28). A few studies have revealed promising results of the GIR technique among Malaysian children, especially in clinical settings (29–31). An academic investigation of the effects of the GIR technique on the psychological well-being of Malaysian children from mainstream primary school settings, especially when the course is delivered fully online, may add a new perspective to this technique in the COVID-19 era.

## Methods

### Research Design, Subject Eligibility and Randomisation

The present study utilised a non-blinded randomised controlled trial (RCT) design among primary school children from a selected school in Kelantan, Malaysia. The school counsellor provided a name list of Standard 5 students as potential participants, whose parents were informed of the project. Thereafter, the researcher organised an online meeting with the parents to invite them and their children to participate in the study. Interested parents were the main caregivers and who were required to stay with their children during the experiment. They declared possessing appropriate electronic devices, such as personal computers, laptops or mobile phones with a stable internet connection. This was crucial to ensure the accessibility of the intervention tool to the participants, as well as enable effective researcher–participant online communication amid the COVID-19 pandemic. Children with no neurodevelopmental issues that could impair learning and cognitive functioning, who were willing to participate and commit in the study activities, had consent of their parents, and who understood basic Bahasa Malaysia, were recruited for the study.

The sample size was calculated using G*Power 3.1 software. Utilising a primary analysis of repeated measures ANOVA, with α error of 5% and power (1-β) of 80%, the total minimum required sample size was 34 children—that is, 17 children per group. Considering potential non-response or a 20% drop-out rate, it was planned to recruit a total of 40 children—20 subjects per group.

**Figure 1:**
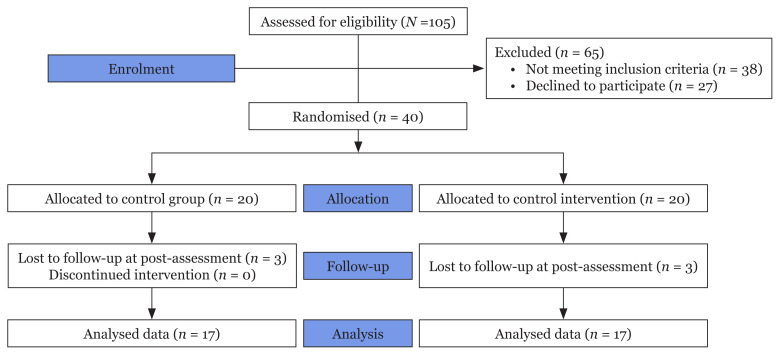
Attrition process of the research subjects in a CONSORT flow diagram

Approvals for the study were obtained from the Ministry of Education, Malaysia; the State Educational Division; the respective school; and the Universiti Sains Malaysia Human Research Ethical Committee. Convenience sampling was implemented to recruit participants and randomisation was utilised to ensure an equal chance of allocation of respondents to either the experimental or control group. Randomisation was performed by a graduate student who was not involved in the research, by executing a random number generator function “= RAND ()” in Microsoft Excel to assign a number for each participant to be assigned either as intervention or control assignment (32). The control group was provided with the audio recording of the GIR technique post-assessment. Information on attrition can be drawn from the CONSORT flow diagram in Figure 1.

### Research Tools

#### The Guided Imagery Relaxation Technique

The GIR technique is an audio-recorded relaxation script developed by one of the authors to induce relaxation in children (31, 33). It involves five relaxing stages: i) induction, ii) deepening, iii) coping strategies, iv) positive suggestions and v) closure. The listeners are recommended to sit comfortably and take a few deep breaths during the induction phase. Their experience of relaxation is enhanced through deep breathing and progressive muscle relaxation instructions. Thereafter, they are introduced to imagery places, where they visualise peace and calmness. They are guided to be aware of, and to experience, physical, mental and emotional relaxation through imagination and utilisation of multiple senses including vision, touch, taste, smell and hearing. Incorporated in the script are recommendations to utilise positive coping strategies, behaviours and cognitive elements to promote hopes, satisfaction and overall well-being. A pilot study utilising this technique has validated the tool and proven that the audio is audible, feasible, comprehensible and relaxation-inducing for Malaysian children. The practice of listening to a series of relaxation audios was reported to reduce children’s emotional distress and anxiety (31).

#### Stress in Children Questionnaire

The Stress in Children (SiC) questionnaire was developed by Osika, Friberg and Wahrborg to assess perceived stress as reported by school children aged 9 years old–11 years old. As many as 21 items measured three factors: i) distress, ii) lack of well-being and iii) lack of social support. The respondents rated the factors on a 4-point Likert scale: ‘0’ for none, ‘1’ for sometimes, ‘2’ for almost always and ‘3’ for always. Scores were evaluated by cumulating all points and dividing by the total number of items to obtain a mean score; higher scores indicated higher degrees of perceived stress. The SiC questionnaire exhibited good internal consistency (ranging from a = 0.61 to 0.86) and strong concurrent validity across studies (34, 35).

**Figure 2:**
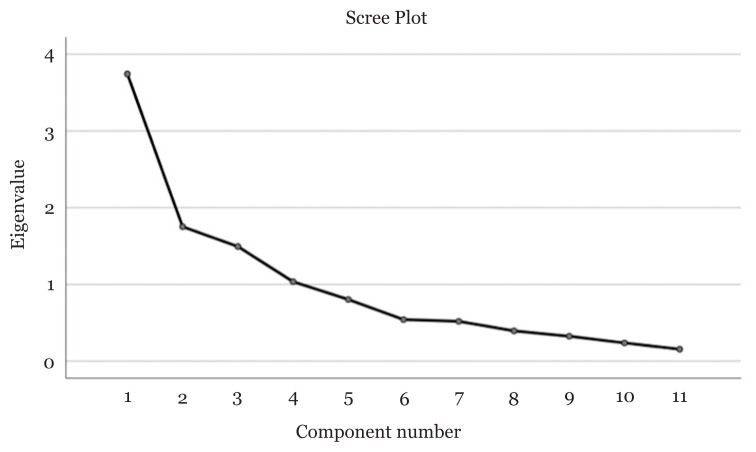
Factor analysis of SiC questionnaire scores

As this questionnaire is not yet available in Bahasa Malaysia, a forward- and backward-translation process was conducted to translate it into Bahasa Malaysia to be used for the target population. A pilot study was conducted to validate the translated scale. The validation of the questionnaire was demonstrated through reliability analysis and factorial validity. Several preliminary analyses were conducted, resulting in the removal of 10 items prior to proceeding with factor analysis on the abridged 11-item revised SiC questionnaire. The Kaiser-Meyer-Olkin (KMO) value measure of sampling adequacy demonstrated a value of 0.693, which was above the minimum threshold of 0.6 and indicated that the sampling was sufficient. Bartlett’s test of sphericity displayed a significant value of less than 0.001, which suggested that the correlation matrix did not produce an identity matrix. Furthermore, the diagonal of the anti-image correlation matrix showed that all values were more than 0.5. As shown in Figure 2 for the factor analysis, the three-factor model demonstrated Eigenvalues of at least 1 and combined accounted for 63.53% of the variance (36). The new, abridged 11-item revised SiC questionnaire was validated via factor analysis and displayed good internal consistency (α = 0.783).

#### Strengths and Difficulties Questionnaire-Parent Report

The Strengths and Difficulties Questionnaire-Parent Report (SDQ-PR) was developed by Goodman to assess psychological well-being of children aged 4 years old–16 years old. There are 25 items measuring five domains: i) conduct problems, ii) emotional symptoms, iii) hyperactivity, iv) peer problems and v) prosocial behaviour. Participants rate their responses on a 3-point Likert scale: ‘0’ for not true, ‘1’ for somewhat true and ‘2’ for certainly true on statements related to their children; five items are reverse-coded. The total scores from the first four domains—excluding prosocial behaviour—are added, with a lower score indicating higher degree of well-being. The original SDQ-PR reported satisfactory internal consistency (α = 0.73) and good convergent validity (*r* = 0.73) with Child Behaviour Checklist (37–39). Although the SDQ-PR is predominantly used for assessing emotional and behavioural difficulties in children, studies have reported that its Bahasa Malaysia version is a valid and reliable instrument to indicate well-being of Malaysian children (40, 41). Thus, it was applied in this study using a version made available by the author in the website - https://www.sdqinfo.org/py/sdqinfo/b3.py?language=Malay (42).

#### Attendance, Tracking and Feedback Forms

An online session is conducted weekly to administer the GIR technique in a group led by the researcher to monitor the children’s progress and challenges and compliance to the GIR daily listening task. Children’s attendance is recorded on an attendance sheet and their compliance with the daily listening task is tracked using a researcher-developed tracking form. The number of entries in the form enable the researcher to track the frequency of the GIR technique practised by the participants each week. Finally, children’s feedback is gathered after each online session using the emoticons feedback cards. These include several cards containing emoticons, representing the emotions of ‘sad’, ‘just okay’ and ‘happy’. Children are asked about their feelings and are required to choose from the cards options. A study suggested that this visual presentation matched 11-year-old children’s understanding to ensure relatively accurate feedback regarding their feelings and experiences of practising the GIR technique (43).

### Procedure

After participant recruitment and randomisation to intervention and control groups, the first online briefing session was conducted through Google Meet to provide detailed information regarding the study and forms to be completed. All forms were prepared using Google forms, and links were shared with the participants. Questions and expectations from the participants were responded to. The pre-assessment survey battery was completed. The children completed the SiC questionnaire and their parents completed the SDQ-PR. Post-assessment occurred soon after the last intervention session, whereby the Google forms links were re-shared with the participants.

**Figure 3:**
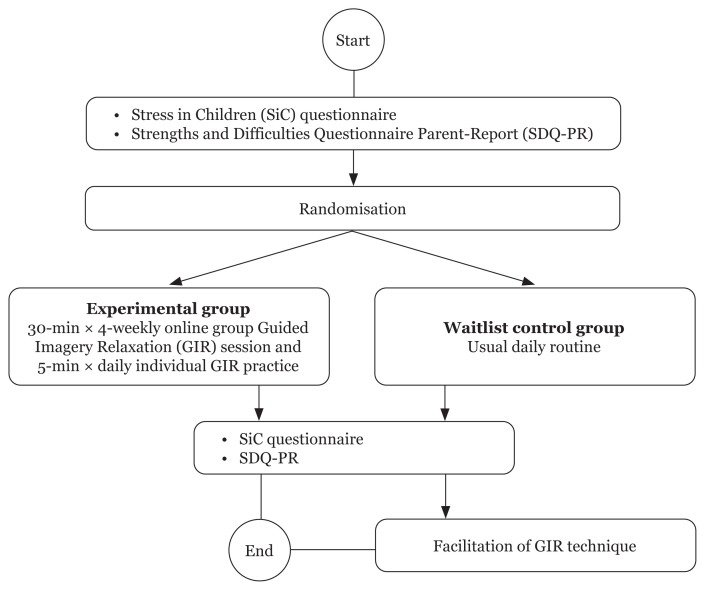
Flow chart for the process of data collection

The children in the intervention group were introduced to the GIR technique and its benefits. They listened to the audio for the first time in an online group and shared their feelings and experiences about it. In the intervention group, they were required to listen to at least 5 min of GIR recorded audio daily throughout the 4 weeks of intervention. Each listening task was recorded in the tracking form. Additionally, they had to attend weekly online sessions where the GIR technique was administered by the researcher, who also monitored the progress of the daily listening task through the tracking form record. Each participant’s subjective feedback was obtained after each weekly online monitoring session. Feedback was gathered verbally during online sessions as the children responded to questions about their feelings, either voluntarily or as cued by the emoticons cards shown by the researchers on screen. Further details of the process are provided in Figure 3.

### Statistical Analysis

The data were analysed using IBM SPSS version 26.0 software. A validation study was conducted to determine the psychometric properties of the SiC questionnaire. Data screening was performed and internal reliability and factorial validity was examined. The questionniare’s reliability was verified using Cronbach’s alpha (α) along with item-total correlation of each item, inter-item correlation and item’s contribution to Cronbach’s alpha.

Descriptive statistics were explored to summarise the socio-demographic characteristics of subjects: continuous data were presented as mean and standard deviation, whereas categorical data were presented as frequency and percentage. Next, reliability analysis and normality tests were conducted on stress and well-being scored via the examination of the values of skewness and the Shapiro-Wilk test prior to running any inferential analyses. Mixed-model ANOVA tested the hypotheses as well as within- and between-interactions. Subsequently, analysis of the participants regarding attending the weekly online monitoring sessions and their regularity of practising the guided imagery relaxation audio were tabulated with frequency and percentage.

## Results

### Demographic and Baseline Information

A total of 34 (*N* = 34) consenting participants were randomised into intervention group (*n* = 17) or control group (*n* = 17). All belonged to either of two Primary 5 classes: Al Biruni or Al Farabi; and were aged 11 years old during the study period. More girls (*n* = 21, 61.8%) joined the study compared to boys (*n* = 13, 38.2%). At baseline, there were no significant differences between intervention and control groups in terms of gender and class, as well as stress and well-being scores. In particular, the mean scores of the SiC questionnaire reported by participants from both groups were equally minimal with a mean of 1.15 (SD = 0.27 for the intervention group and SD = 0.22 for the control group). The mean scores of the SDQ-PR in the intervention and control groups were 10.47 (SD = 4.88) and 8.53 (SD = 4.53), respectively. Both scores were classified as ‘normal’ level of well-being, implying that, in general, the participants did not have significant levels of stress pre-intervention. The results are presented in Table 1.

### Normality Tests

The summary table of normality tests for Shapiro-Wilk and skewness is displayed in Table 2. For the current study, the Shapiro-Wilk test was used as the numerical means of assessing normality for its appropriateness for sample sizes smaller than 50 (44). The test showed a non-significant deviation from normal distribution, with *P*-values for both variables: stress and well-being across different time of measurement and different treatment groups greater than 0.05. This indicated that the null hypothesis was to be accepted and data obtained were normally distributed (45). Next, data skewness was examined as it is the measure for a variable’s asymmetry of distribution; if the absolute z-scores for skewness are above 1.96, the null hypothesis is rejected and distribution is non-normal (46). As shown in Table 2, all the z-values are below 1.96 regardless of being positive or negative; thus, the data obtained are normally distributed.

### The GIR Technique’s Effects on Stress and Well-Being

To investigate the respective significant difference of stress and well-being for between-group of subjects across pre- and post-interventions, mixed-model ANOVA was performed on both variables. For stress, a 2 (Time) × 2 (Group) mixed-model ANOVA revealed that the main effect of the treatment group was not significant through *F* (1, 32) = 0.36; *P* > 0.05, partial *η*^2^ = 0.01. Additionally, a non-significant relationship between Time × Group was obtained through *F* (1, 32) = 0.82; *P* > 0.05; partial *η*^2^ = 0.03. Regarding well-being, the 2 (Time) × 2 (Group) mixed-model ANOVA revealed that the main effect of the treatment group on well-being in general was not significant through *F* (1, 32) = 1.85; *P* > 0.05; partial *η*^2^ = 0.06. Moreover, a non-significant association between Time × Group was also demonstrated through *F* (1, 32) = 0.20; *P* > 0.05; partial *η*^2^ = 0.01. Further details are provided in Tables 3 and 4.

### Attendance and Tracking Form

Within the intervention group, 11 (64.7%) participants achieved full attendance, implying they attended all four online GIR monitoring sessions as well as briefing and debriefing sessions. Two participants (11.8%) attended three sessions; three (17.6%) attended two sessions; and one (5.9%) attended one session. Of the 17 students in the active treatment group, eight (47.1%) completed the tracking form and practiced the given GIR technique in audio format for 5 min–10 min daily, that is, they played the audio once or twice daily. The remaining nine (52.9%) did not complete the tracking form and did not listen to the GIR technique audio on a daily basis because of various unspecified reasons.

## Discussion

The results indicate that the children in the intervention group did not report significant changes in their level of stress and psychosocial well-being, which is contrary to a few previous findings (47–49). The GIR technique is considered a relaxing tool that encourages better self-control, social cooperation, and emotional and behavioural development (50, 51). Through this technique, children may gain better insight to restructure past negative experiences. Additionally, they are assumed to have higher levels of creativity and imagination to solve problems, which could lead to reduced stress and improved sense of well-being (52–54).

Nevertheless, the present finding is consistent with that of Nilsson et. al (55), who noted that the GIR technique was not necessarily applicable for, or helpful to, each child who practiced it. Another study suggested that this technique exerted more and higher intensity of positive effects on children with psychological symptoms than on those with normal mental health, who were the target participants in the current study (56). Although positive developments could be noticed at the beginning stage, the effects of the GIR technique may begin to subside after 3–4 weeks of intervention (57). Possibly, the effects of this technique on stress and well-being may appear to be insignificant in the present study after weeks of intervention. The insignificant findings could also be discussed from different perspectives, as follows.

### Time Availability

The study was conducted during the COVID-19 pandemic with the operation of the Movement Control Order (MCO) in the country when children attended online classes. Although the GIR technique requires merely 5 min–10 min of daily practice, the children had daily online class—a few even received plentiful homework. Adjusting to the challenges of schooling from home, they had to cope with significant distress because of physical inactivity and lack of social contact with peers (58). The children admitted they did not have adequate time to practice the GIR technique or that they forgot about it (59, 60). Time constraints and lack of motivation may indeed cause insignificant effects on the outcome variables measured (61).

### Drop-Out and Non-Adherence

The adherence rate was 64.7%, which is not uncommon for interventional studies because participation and retention of participants is a challenge even for a comparatively brief study duration. Unsatisfactory drop-out and adherence rates imply low effectiveness of virtually monitored interventions (62, 63). Regular practice will enable participants to become more skillful and competent in relaxation, thus improving their general well-being (64, 65).

### Internet Accessibility and Connectivity

According to a written parliamentary reply released by the Minister of Education in July 2020, 36.9% of students nationwide did not possess any, or adequate, device(s) to attend online classes. Many children had to share devices with their siblings or parents. Even when they did have appropriate devices, the internet connection could be unstable or weak because of poor infrastructure and geographical factors (66, 67). Problems such as poor or unstable online connectivity were observed during our monitoring sessions. This could result in increased distress among participants and cause frustration in them (68).

### Physical Versus Virtual Contact

The intervention in the current study was delivered in a digital, online format. The content was shared using different modalities, such as presentation slides, mindfulness audio and online face to face interactive sessions. Although virtual-based mindfulness interventions have been effective in improving psychological well-being and reducing stress, their effects are relatively short-lived and not as significant (63, 69–71). To date, they have not been found to be as effective as face-to-face interventions for enhancing mental health (72). In fact, digital delivery may undermine the effectiveness of intervention and probability of enhancing the outcomes. This could possibly be attributed to the difficulty of developing a trusted, positive therapeutic alliance with the practitioners (61).

## Conclusion

Contrary to our hypothesis, the children in the intervention group, to whom the GIR technique was delivered online, did not report significant changes in the sense of stress and well-being compared to the control group. Nevertheless, the current project could be the first local study on the effects of the Malay version of the GIR technique on stress and well-being of primary school children over online-based delivery and monitoring. While many studies have investigated the negative impacts of COVID-19 on adults, only a few have explored its impact on children (73). The special needs of children in the midst of epidemics or disastrous events are often ignored: “What kind of experience studying from home represents for children?” or “How could the family members cooperate to reduce the negative psychological impacts for children in this period of fear and uncertainty?” (74). The current study could encourage more investigation into the potential mental health problems and well-being of children (75). Additionally, it could induce adults to envisage strategies for the positive growth and development of children (76).

This study has a few limitations. A relatively small sample size of participants who successfully completed the study may impact the findings. Moreover, the withdrawal of participants from the study may have affected the power of the study, resulting in insignificant results. Therefore, a larger sample may be helpful in future studies. Additionally, issues of generalisability and bias owing to the design limitation of the research methodology were present. Furthermore, virtual-based monitored interventions are affected by participants’ adherence and internet connectivity issues; future studies should attempt to overcome these challenges (77).

In the near future, mindfulness-based approaches should be continuously modified and strive to become a promising intervention modality for primary school children. Meanwhile, substantial room for improvement and revision exists for the implementation and delivery of the Malay-translated GIR technique (78).

## Figures and Tables

**Table 1:** Brief demographic dan baseline information of participants by groups (*N* = 34)

Characteristics	Intervention (*n = 17*)		Control (*n = 17*)		Test statistics^a^	
		
	*n*	%	*n*	%	*χ* *^2^*	*P*-value
Gender					1.09	0.30
Male	5	29.41	8	47.06		
Female	12	70.59	9	52.94		
Class					0.45	0.51
5 Al-Biruni	7	41.18	9	52.94		
5 Al-Farabi	10	58.82	8	47.06		

**Continuous variables**	**Mean (SD)**		**Mean (SD)**		**Test statistics**	

Stress (SiC)	1.15 (0.27)		1.15 (0.22)		*t* (33) = 0.00, *P* = 1.00	
Well-being (SDQ-PR)	10.47 (4.88)		8.53 (4.53)		*t* (33) = 1.45, *P* = 0.24	

Notes: Degree of freedom (*df*) for chi-squared tests, *df* = 1;

a Chi-squared test and *P*-values were reported using Fisher’s exact test

**Table 2:** Summary table for tests of normality: Shapiro-Wilk and skewness

Variables	Time points	Group	Shapiro-Wilk			Skewness

			Statistic	df	*P-*value	
Stress	Pre	Intervention	0.92	17	0.17	0.91
		Control	0.95	17	0.40	0.23
	Post	Intervention	0.96	17	0.67	−0.18
		Control	0.94	17	0.37	0.65
Well-being	Pre	Intervention	0.98	17	0.94	0.56
		Control	0.94	17	0.37	0.65
	Post	Intervention	0.90	17	0.07	1.39
		Control	0.94	17	0.29	0.71

**Table 3:** Descriptive statistics of stress and well-being across treatment groups and time of measurement

Variable	Group	Time	Mean (SD)	95% Confidence interval	

				Lower bound	Upper bound
Stress	Intervention	Pre	1.15 (0.26)	1.03	1.27
		Post	1.19 (0.31)	1.05	1.33
	Control	Pre	1.15 (0.22)	1.03	1.27
		Post	1.10 (0.25)	0.96	1.24
Well-being	Intervention	Pre	10.47 (4.88)	8.15	12.80
		Post	11.12 (5.84)	8.48	13.76
	Control	Pre	8.53 (4.53)	6.21	10.85
		Post	8.65 (4.78)	6.01	11.28

**Table 4:** Stress and well-being within and between subjects across pre- and post-intervention

Variable	Source	SS^a^	*df* b	MS	*F*	*P*-value	Partial *η*^2^
Stress	Intercept	89.68	1	89.68	913.64	0.000	0.97
	Group	0.04	1	0.04	0.36	0.554	0.01
	Error	3.14	32	0.10			
Well-being	Intercept	6,386.49	1	6,386.49	143.09	0.000	0.82
	Group	82.72	1	82.72	1.85	0.183	0.06
	Error	1,428.29	32	44.63			

Notes:

a SS = sum of squares;

b Degrees of freedom (*df*)
